# Scutellariae Radix and Citri Reticulatae Pericarpium Mixture Regulate PPAR*γ*/RXR Signaling in Reflux Esophagitis

**DOI:** 10.1155/2022/6969241

**Published:** 2022-01-04

**Authors:** Jin A. Lee, Mi-Rae Shin, Hae-Jin Park, Seong-Soo Roh

**Affiliations:** ^1^Department of Herbology, College of Korean Medicine, Daegu Haany University, 136 Shinchendong-Ro, Suseong-Gu, Deagu 42158, Republic of Korea; ^2^DHU Bio Convergence Testing Center, 1 Hanuidae-Ro, Gyeongsan 38610, Republic of Korea

## Abstract

**Objective:**

Gastroesophageal reflux disease (GERD) is a gastrointestinal disorder in which stomach contents reflux into the esophagus, causing complications such as mucosal damage. GERD is a very common disease and is on the rise worldwide. The aim of this study was to assess the impact of a Scutellariae Radix and Citri Reticulatae Pericarpium mixture (SC) on esophageal mucosal injury in rats with chronic acid reflux esophagitis (CARE).

**Methods:**

After inducing reflux esophagitis through surgery, the group was separated and the drug was administered for 2 weeks: normal rats (Normal, *n* = 8), CARE-induced rats were treated with distilled water (Control, *n* = 8), CARE-induced rats were treated with vitamin E 30 mg/kg body weight (VitE, *n* = 8), CARE-induced rats were treated with SC 100 mg/kg body weight (SC100, *n* = 8), and CARE-induced rats were treated with SC 200 mg/kg body weight (SC200, *n* = 8).

**Results:**

SC treatment significantly reduced the degree of esophageal mucosal damage, significantly reduced levels of MDA and MPO, and inhibited the activation of the NF-*κ*B inflammatory pathway by activating the PPAR*γ*/RXR pathway. In addition, SC treatment significantly regulated the expression of arachidonic acid-related proteins (COX-1, COX-2, and PGE_2_) and modulated the MMP/TIMP proteins in reflux esophagitis.

**Conclusion:**

Consequently, SC improved the damage to the esophageal mucosa. Also, the anti-inflammatory effects of the SC suggested the inhibition of NF-*κ*B pathway through the activation of the PPAR*γ*/RXR pathway, thereby reducing the expression of inflammation-related cytokines.

## 1. Introduction

Gastroesophageal reflux disease (GERD) is defined as a disease in which gastric contents reflux into the esophagus and occurs when the reflux of contents causes problematic symptoms and complications [1]. GERD is caused by stress, intake of overeating, high-fat diet, and alcohol, and symptoms such as abdominal bloating, indigestion, chest pain, burning sensation, and heartburn appear. Nighttime heartburn causes sleep disturbance, and sleep deprivation and stress affect your life the next day [2, 3].

In a previously published study, it was reported that peroxisome proliferator-activated receptors (PPARs) are involved in the regulation of immune response and inflammation in gastrointestinal inflammation, and play an important role as a therapeutic target for gastrointestinal inflammation management [4]. PPAR is activated by heterodimerization with retinoid X receptor (RXR), and the activated PPAR/RXR heterodimers induce the transcription of a target gene [5, 6]. PPAR regulates the expression of antioxidants and oxidative promoters against oxidative stress caused by inflammation, and it regulates the activation of nuclear factor-*κ*B (NF-*κ*B), a transcription factor mediating the inflammatory response [7, 8]. Thus, PPAR exerts anti-inflammatory effects by regulating the expression of antioxidants and NF-*κ*B in esophageal inflammation [9].

Scutellariae Radix (Hwagngeum in Chinese) is the dried root of plants of the genus Lamiaceae, and in traditional Korea medicine (TKM), Scutellariae Radix is known to have the function of removing heat and moisture and detoxifying [10]. Scutellariae Radix has various flavonoid components such as baicalin and baicalein, and these components show great effects in the treatment of inflammation and cancer-related diseases [11]. Citri Reticulatae Pericarpium (Chenpi in Chinese) is the dried peel of fruit of the Citrus reticulata Blanco, and in TKM, Citri Reticulatae Pericarpium is known to have the function of removing moisture and tonifying spleen [12, 13]. In modern medicine, Citri Reticulatae Pericarpium has been proven to have antiulcer and anti-inflammatory effects [14], and it is known that it can effectively inhibit DNA damage through its antioxidant ability [15].

The aim of this study was to determine whether a Scutellariae Radix and Citri Reticulatae Pericarpium mixture (SC) could alleviate esophageal mucosal injury as a result of chronic acid reflux esophagitis. As a result of the previous study, it was confirmed that SC alleviated the damage to the esophageal mucosa in acute reflux esophagitis [16], and furthermore, the mechanism for the effect in chronic reflux esophagitis was confirmed.

## 2. Materials and Methods

### 2.1. Materials

Vitamin E, 2-thiobarbituric acid, 1,1,3,3-tetramethoxypropane, and phenyl methyl sulfonyl fluoride (PMSF) were provided by Sigma-Aldrich, Merck KGaA. Phosphoric acid was purchased from Duksan Company. The protease inhibitor mixture solution and EDTA were provided by Wako Pure Chemical Industries, Ltd. The Pierce BCA protein assay kit was provided by Thermo Fisher Scientific, Inc. Enhanced chemiluminescence reagent (ECL), western blotting detection reagents, and pure nitrocellulose membranes were obtained from GE Healthcare. NADPH oxidase 2 (NOX2; SC-130543), p47^phox^ (SC-17845), peroxisome proliferator-activated receptor *γ* (PPAR*γ*; SC-7273), retinoid *X* receptor (RXR; SC-774), superoxide dismutase (SOD; SC-17767), catalase (SC-271803), nuclear factor kappa B p65 (NF-*κ*Bp65; SC-8008), inhibitor of kappa B alpha (I*κ*Bɑ; SC-1643), phospho-inhibitor of kappa B alpha (p-I*κ*Bɑ; SC-8404), tumor necrosis factor alpha (TNF-ɑ; SC-133192), interleukin 6 (IL-6; SC-57315), cyclooxygenase-1 (COX-1; SC-19998), cyclooxygenase-2 (COX-2; SC-19999), matrix metallopeptidase-2 (MMP-2; SC-13595), matrix metallopeptidase-9 (MMP-9; SC-13520), tissue inhibitor of metalloproteinase-1 (TIMP-1; SC-21734), and tissue inhibitor of metalloproteinase-2 (TIMP-2; SC-21735) were purchased from Santa Cruz Biotechnology, Inc. Prostaglandin E2 (PGE2; ab2318) was purchased from Abcam. Goat anti-rabbit and goat anti-mouse immunoglobulin G (IgG) horseradish peroxidase (HRP)-conjugated secondary antibodies were purchased from GeneTex, Inc. Zoletil®50 was purchased from Virbac Laboratory, and Isotroy was purchased from Troikaa Pharmaceuticals, Ltd.

### 2.2. Preparation of the Plant Material

Scutellariae Radix and Citri Reticulatae Pericarpium were purchased from OMNIHERB CO., LTD. Extracts of the dried Scutellariae Radix (200 g) and Citri Reticulatae Pericarpium (200 g) were obtained by the addition of 10X volume of boiled water at room temperature (2 h for each extraction), and the solvent was evaporated in vacuo to obtain powders (Scutellariae Radix, 29%; Citri Reticulatae Pericarpium, 35%). The two prepared powders were stored at −80°C. If necessary, Scutellariae Radix and Citri Reticulatae Pericarpium were mixed in a 1 : 1 ratio to prepare a mixture (Scutellariae Radix and Citri Reticulatae Pericarpium mixture; SC) and then dissolved in water for use.

### 2.3. Scutellariae Radix and Citri Reticulatae Pericarpium Analysis by HPLC Chromatogram

The Scutellariae Radix extract (10 mg) was dissolved in 10 mL of 50% methanol. We injected 20 *μ*L of the sample into a reverse-phase high-performance liquid chromatograph (HPLC) using a Phenomex Gemini NX C18 (4 : 6 150 mm, 3-*μ*m pore size), with a column temperature of 35 C. The solvent system used was given as follows: solvent A (1% formic acid), solvent B (acetonitrile); A : B = 100 : 00 (0 min) ⟶ 100 : 0 (3 min) ⟶ 90 : 10 (5 min) ⟶ 90 : 10 (7 min) ⟶ 80 : 20 (12 min) ⟶ 70 : 30 (17 min) ⟶ 70 : 30 (22 min) ⟶ 40 : 60 (31 min) ⟶ 40 : 60 (35 min) ⟶ 5 : 95 (40 min) ⟶ 50 : 50 (45 min) at a flow rate of 0.6 mL/min with a UV absorption monitoring at 277 nm. The peaks of baicalin and baicalein were assigned by comparison of retention times and UV spectra of authentic standards.

The extract of Citri unshius pericarpium (0.5 mg) was dissolved in 10 mL of 100% methanol. We injected 1 *μ*L of the sample into a Waters Acquity UPLC system (Waters®, Milford, MA, USA) with a reversed-phase C18 column (Phenomenex 2.6 *μ*m C18 100 Å, 2.1 × 100 mm, Phenomenex, Torrance, CA, USA). The solvent system used was as follows: solvent A (deionized water with 0.1% formic acid), solvent B (100% acetonitrile with 0.1% formic acid); A : B = 82 : 18 (0 min) ⟶ 82 : 18 (1 min) ⟶ 75 : 25 (15 min) ⟶ 0 : 100 (20 min) ⟶ 0 : 100 (25 min) ⟶ 82 : 18 (26 min) ⟶ 82 : 18 (32 min) at a flow rate of 0.2 mL/min with a UV absorption monitoring at 284 nm. The peaks of narirutin and hesperidin were assigned by comparison of retention times and UV spectra of authentic standards.

### 2.4. Induction of Chronic Acid Reflux Esophagitis and Treatment

The animal experiment protocol was performed with the approval of the Animal Care and Use Committee of Daegu Haany University (Approval No. DHU2021-087). The 5-week-old male Sprague Dawley rats (body weight, 130–150 g) were obtained from DBL (Eumseong, Korea). After 1-week adaptation (environmental conditions; to 12 h light/dark cycle, controlled temperature (22 ± 2°C), and humidity (50 ± 5%)), surgery to induce reflux esophagitis was performed in all rats except the normal group (*n* = 8). First of all, anesthesia was induced in rats using tiletamine and zolazepam (Zoletil®50; 37.5 mg/kg). Afterwards, the transitional region (i.e., limiting ridge) between the forestomach and the glandular portion of the stomach was ligated with 2–0 black silk thread in order to restrict the compliance of the stomach. Also, a latex ring (18-Fr Nelaton catheter) was placed at siting the pyloric sphincter [17, 18]. After surgery, gentamicin and dexamethasone were injected for 4 days to prevent infection (subcutaneous injection), and rats were given water after 24 h and ingested feed after 48 h. Rats had surgical recovery and induction period for 7 days after surgery. After that, rats were divided into 5 groups (*n* = 8 per group) as follows: (i) Normal, normal group; (ii) Control, CARE-induced rats were treated with distilled water; (iii) VitE, CARE-induced rats were treated with vitamin E (30 mg/kg body weight); (iv) SC100, CARE-induced rats were treated with SC (100 mg/kg body weight); and (v) SC200, CARE-induced rats were treated with SC (200 mg/kg body weight).

After group separation, body weight and food intake were measured for 14 days, and drugs were orally administered. On the 15th day, rats were anesthetized by Isotroy inhalation anesthesia (induction, 4% isoflurane; maintenance, 2% isoflurane) for 5–7 min and sacrificed by inhalation anesthesia (isoflurane, Telangana, India), and blood and esophageal tissue were collected.

### 2.5. Esophageal Ulcer Ratio

After sacrifice, the esophagus of the rat was cut from the gastroesophageal junction to the pharynx after sacrifice. The dissected esophagus was taken using an optical digital camera and then analyzed using the I-Solution Lite software program (InnerView Co., Korea). The gross mucosal ulcer ratio (%) = (width of area withwidth of area with esophageal mucosal ulcer /width of total area >  of esophagus) × 100.

### 2.6. Measurement of MDA and MPO Levels

The malondialdehyde (MDA) levels were measured according to the method of Mihara and Uchiyama [19]. 1,1,3,3-Tetramethoxypropane was used as a standard sample. After mixing the sample and 1% phosphoric acid, 0.67% thiobarbituric acid was added and boiled for 45 min at 95°C. After that, butanol was mixed and centrifuged (3,000 rpm, 10 min) to obtain the supernatant. The supernatant was dispensed, and absorbance was measured at 540 nm using a UV-VIS spectrophotometer. Also, myeloperoxidase (MPO) was measured using an assay kit (Cat. K744) purchased from BioVision (Milpitas, CA, USA).

### 2.7. Preparation of Nuclear and Cytosol Factions

The extraction of protein was performed as described by Komatsu [20]. For cytosol fractions, esophageal tissues were homogenized with 250 mL ice-cold lysis buffer A containing 10 mM HEPES (pH 7.8), 10 mM KCl, 2 mM MgCl_2_, 1 mM DTT, 0.1 mM EDTA, 0.1 mM PMSF, and 1,250 *μ*L protease inhibitor mixture solution. The tissue homogenates were incubated (4°C for 30 min), and then, 10% NP-40 was mixed well. After centrifugation (12,000 rpm at 4°C for 2 min) using Eppendorf 5415R (Hamburg, Germany), the supernatant (cytosol fractions) was separated using new Eppendorf tubes. The pellets were washed twice with the lysis buffer, and the supernatant was discarded. After that, the pellets were suspended with 20 mL ice-cold lysis buffer C containing 300 mM NaCl, 50 mM HEPES (pH 7.8), 50 mM KCl, 1 mM DTT, 0.1 mM PMSF, 0.1 mM EDTA, 1% (v/v) glycerol, and 100 *μ*L protease inhibitor mixture solution suspended and incubated (4°C for 30 min). After centrifugation (12,000 rpm at 4°C for 10 min), the supernatant (nuclear fractions) was collected in new tubes. Both cytosol and nuclear fractions were stored at −80°C before the analysis.

### 2.8. Immunoblotting Analyses

For the estimation of NOX2/p47^phox^/PPAR*γ*/RXR/SOD-1/catalase/NF-*κ*Bp65/p-I*κ*B*α*/I*κ*B*α*/TNF-*α*/IL-6/COX-1/COX-2/PGE_2_/MMP-2/MMP-9/TIMP-1/TIMP-2/*β*-actin/histone (1 : 1000), 12 *μ*g of proteins was electrophoresed through 8–12% sodium dodecyl sulfate-polyacrylamide gel (SDS-PAGE). Separated proteins were transferred to a nitrocellulose membrane, blocked with 5% (w/v) skim milk solution for 1 h, and then incubated with primary antibodies overnight at 4°C. After the blots were washed, they were incubated with anti-rabbit or anti-mouse IgG HRP-conjugated secondary antibody (1 : 3000) for 2 h at room temperature. Each antigen-antibody complex was visualized using ECL western blotting detection reagents and detected by chemiluminescence with Sensi-Q 2000 ChemiDoc (Lugen Sci Co., Ltd., Gyeonggi-do, Korea). Band densities were measured using ATTO Densitograph Software (ATTO Corporation, Tokyo, Japan) and quantified as the ratio to histone or *β*-actin. The protein levels of the groups are expressed relative to those of the normal rat (represented as 1) [21].

### 2.9. Histological Examination

Histological microscopic examination was performed to evaluate the separated esophagus tissues. The separated esophagus was fixed through a 10% neutral-buffered formalin and embedded in paraffin, and cut into 2 *μ*m sections and stained using hematoxylin and eosin (H&E) for microscopic evaluation. The stained slices were observed under an optical microscope and then analyzed using the I-Solution Lite software program (InnerView Co., Seongnam-si, Gyeonggi-do, Korea).

### 2.10. Statistical Analysis

*In vivo* values were expressed as means ± SD. Statistical comparisons were analyzed by one-way ANOVA tests followed by the least significant difference (LSD) test using SPSS (version 25.0, IBM, Armonk, NY, USA). The values of *P* < 0.05 were considered significant.

## 3. Results

### 3.1. Scutellariae Radix and Citri Reticulatae Pericarpium Analysis by HPLC Chromatogram

It is one of the Korean medicines known for its excellent effect on inflammation of Scutellariae Radix [22]. Flavone components, baicalin and baicalein, are well known as the main components of Scutellariae Radix, and these components show great potential in the treatment of inflammation, cancer, and virus-related diseases [11, 23]. Also, narirutin and hesperidin are the most common flavanone glycosides found in CRP [24]. Narirutin and hesperidin were found to have anti-inflammatory effects, and it was reported in clinical practice that hesperidin reduced inflammatory markers [25, 26]. The contents of baicalin and baicalein in gold used in this experiment were 148 mg/g and 12 mg/g, respectively. Also, the narirutin and hesperidin contents in the dermis were 12 mg/g and 10 mg/g (Supplementary 1), respectively.

### 3.2. Esophageal Lesion Ratio

**Figure 1:**
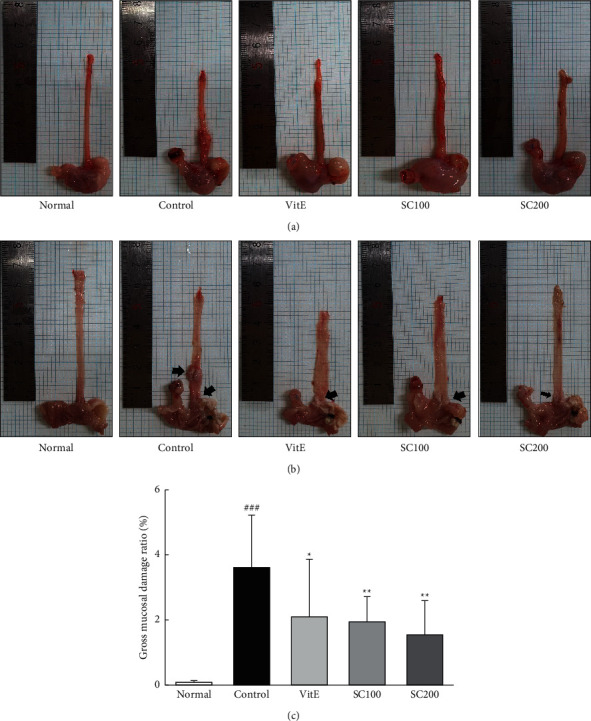
Esophagus tissue damage. A representative gross image of surgical induction of CARE (a); the opened gross esophageal ulcer (b); esophageal ulcer ratio (c). Normal: normal group; control: CARE-induced rats were treated with distilled water; VitE: CARE-induced rats were treated with vitamin E 30 mg/kg body weight; SC100: CARE-induced rats were treated with SC 100 mg/kg body weight; SC200: CARE-induced rats were treated with SC 200 mg/kg body weight. Data are mean ± SD (*n* = 8). Significance: ^###^*P* < 0.001 vs. normal group and ^*∗*^*P* < 0.05, ^*∗∗*^*P* < 0.01 vs. control group.

The gross observation of the esophagus was used to determine the efficacy of SC on the surgically induced CARE lesions. As shown in Figure 1, the Normal group did not exhibit any definite damage of the esophageal mucosa, whereas the esophagus in the Control group showed notable changes in the morphology such as erosions and ulcer. In addition, esophagus in the SC-treated groups decreased significantly tissue damage as erosions and ulcer.

### 3.3. Measurement of MDA and MPO Levels

Serum and tissue analysis was used to detect the MDA and MPO levels, as an indicator of oxidative stress and inflammation. As shown in Table 1, the Control group showed significantly higher MDA levels in serum and tissue compared with the Normal group (serum; 2.27 ± 0.23 vs. 6.37 ± 1.14 nmol/mL, tissue; 1.65 ± 0.11 vs. 2.99 ± 0.38 nmol/mg protein), whereas the levels were significantly reduced in the SC-treated groups. The MPO level was also significantly increased in the Control group compared to the Normal group (1,852 ± 134 vs. 2,849 ± 53 mU/mL), and the increase in these parameters was significantly reduced in the SC-treated groups.

### 3.4. Expression of NADPH Oxidase Proteins

**Figure 2:**
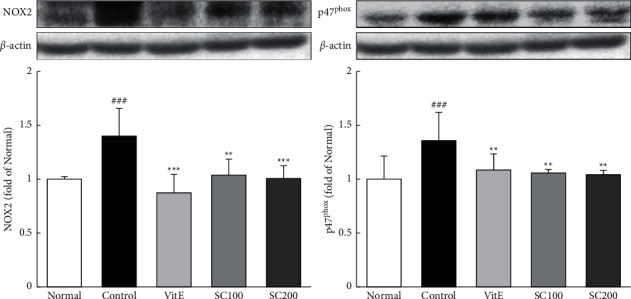
Effects of SC on NADPH oxidases. The expressions of NADPH oxidase were measured by western blotting. Normal: normal group; Control: CARE-induced rats were treated with distilled water; VitE: CARE-induced rats were treated with vitamin E 30 mg/kg body weight; SC100: CARE-induced rats were treated with SC 100 mg/kg body weight; SC200: CARE-induced rats were treated with SC 200 mg/kg body weight. Data are mean ± SD (*n* = 8). Significance: ^###^*P* < 0.001 vs. normal group and ^*∗∗*^*P* < 0.01, ^*∗∗∗*^*P* < 0.001 vs. control group.

The change of ROS-generating NADPH oxidase proteins such as NOX2 and p47^phox^ was examined using western blotting. The protein expressions of NADPH oxidases in the Control group were significantly increased compared with the Normal group (NOX2, 1.40-fold; p47^phox^, 1.36-fold). Conversely, SC treatment significantly decreased their levels. Also, here, treatment with SC reduced their levels to that observed in the Normal group (Figure 2).

### 3.5. Expression of PPAR*γ*/RXR Pathway

**Figure 3:**
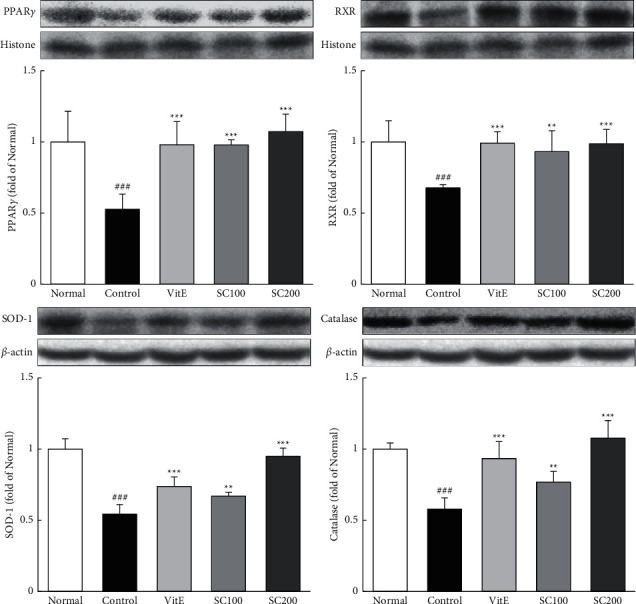
Effects of SC on PPAR*γ*/RXR pathway. The expressions of PPAR*γ*/RXR pathway were measured by western blotting. Normal: normal group; Control: CARE-induced rats were treated with distilled water; VitE: CARE-induced rats were treated with vitamin E 30 mg/kg body weight; SC100: CARE-induced rats were treated with SC 100 mg/kg body weight; SC200: CARE-induced rats were treated with SC 200 mg/kg body weight. Data are mean ± SD (*n* = 8). Significance: ^###^*P* < 0.001 vs. normal group and ^*∗∗*^*P* < 0.01, ^*∗∗∗*^*P* < 0.001 vs. control group.

PPAR*γ*/RXR not only mediates the expression of antioxidant enzymes, but also promotes the inactivation of NF-*κ*B [7, 27]. The expression of PPAR*γ*/RXR and antioxidant enzyme is shown in Figure 3. The protein expressions of PPAR*γ*/RXR and antioxidant enzyme in the Control group were significantly decreased compared with the Normal group (PPAR*γ*; 0.53-fold, RXR; 0.68-fold, SOD-1; 0.54-fold, catalase; 0.58-fold). Conversely, SC treatment significantly increased their levels. In particular, SC treatment increased levels of PPAR*γ* and RXR to that observed in the Normal group, also, the SC200 group increased levels of SOD-1 and catalase observed in the Normal group.

### 3.6. Expression of NF-*κ*B Pathway

**Figure 4:**
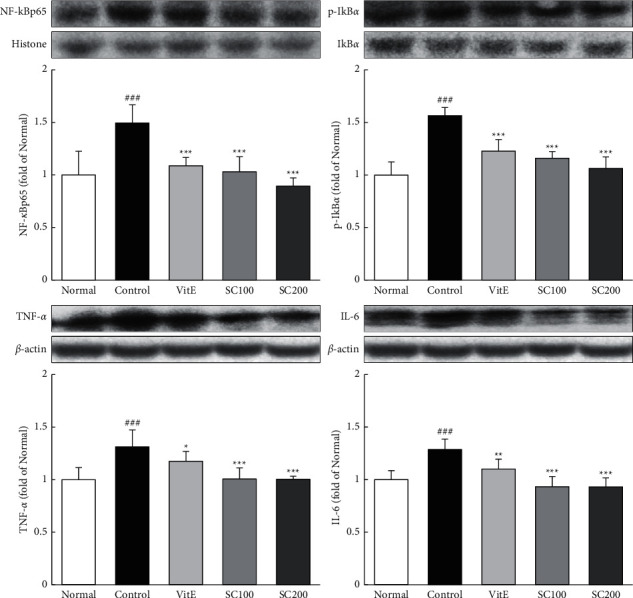
Effects of SC on NF-*κ*B pathway. The expressions of NF-*κ*B pathway were measured by western blotting. Normal: normal group; Control: CARE-induced rats were treated with distilled water; VitE: CARE-induced rats were treated with vitamin E 30 mg/kg body weight; SC100: CARE-induced rats were treated with SC 100 mg/kg body weight; SC200: CARE-induced rats were treated with SC 200 mg/kg body weight. Data are mean ± SD (*n* = 8). Significance: ^###^*P* < 0.001 vs. normal group and ^*∗*^*P* < 0.05, ^*∗∗*^*P* < 0.01, ^*∗∗∗*^*P* < 0.001 vs. control group.

The expression of inflammation-related proteins, including NF-*κ*Bp65, p-I*κ*B*α*, TNF-ɑ, and IL-6, was examined. As shown in Figure 4, the protein expression of inflammation-related proteins were increased in the Control group compared with the Normal group (NF-*κ*Bp65, 1.49-fold; p-I*κ*B*α*, 1.56-fold; TNF-ɑ, 1.31-fold; IL-6, 1.29-fold). However, these increased levels were significantly increased in the SC-treated rats compared with the Control group. In particular, the SC200 group increased levels observed in the Normal group.

### 3.7. Expression of Arachidonic Acid Proteins

**Figure 5:**
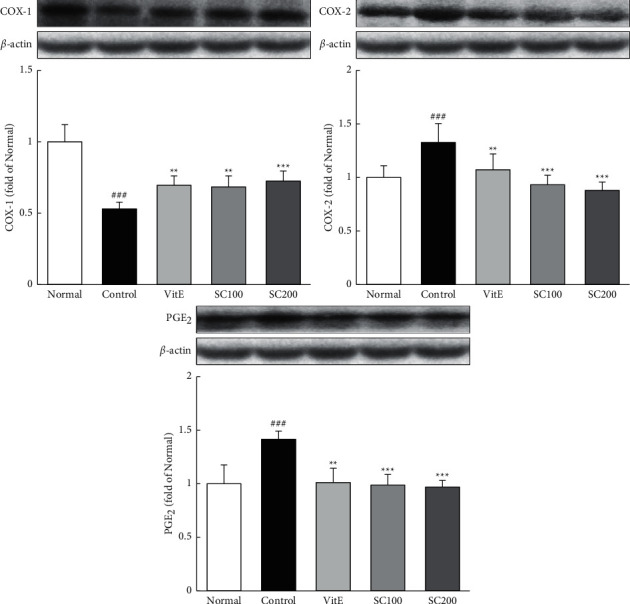
Effects of SC on arachidonic acid-related proteins. The expressions of arachidonic acid protein were measured by western blotting. Normal: normal group; Control: CARE-induced rats were treated with distilled water; VitE: CARE-induced rats were treated with vitamin E 30 mg/kg body weight; SC100: CARE-induced rats were treated with SC 100 mg/kg body weight; SC200: CARE-induced rats were treated with SC 200 mg/kg body weight. Data are mean ± SD (*n* = 8). Significance: ^###^*P* < 0.001 vs. normal group and ^*∗∗*^*P* < 0.01, ^*∗∗∗*^*P* < 0.001 vs. control group.

Prostaglandins are involved in the regulation of the mucous membrane and function of the gastrointestinal tract [28].  Figure 5 shows the expressions of COX proteins, which is known to aid in the production of prostaglandins from arachidonic acid. The expression of COX-1 was decreased in the Control group compared with the Normal group (0.53 ± 0.05), whereas it was significantly increased in the SC-treated rats compared with the Control group. Also, the expression of COX-2 and PGE_2_ was increased in the Control group compared with the Normal group (COX-2; 1.33 ± 0.18, PGE_2_; 1.42 ± 0.08). Conversely, SC treatment significantly decreased levels of COX-2 and PGE_2_. Here, COX-2 and PGE2 levels were reduced to below that of the Normal group by the SC-treated.

### 3.8. Expression of MMP/TIMP Proteins

**Figure 6:**
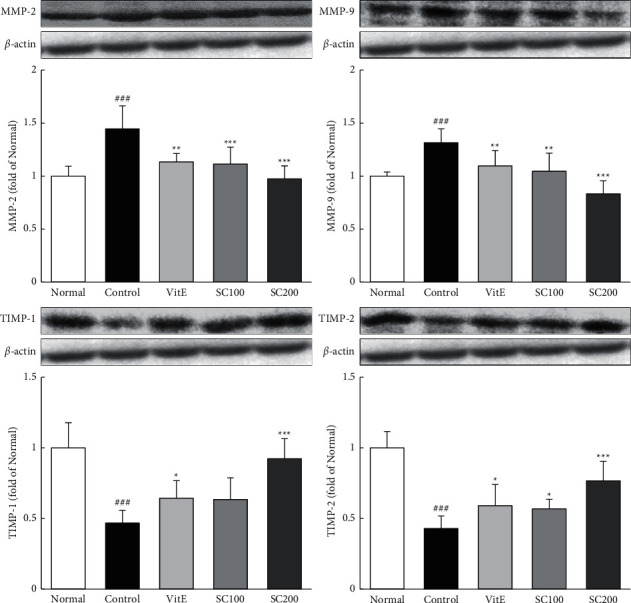
Effects of SC on MMP/TIMP protein. The expressions of MMP/TIMP protein were measured by western blotting. Normal: normal group; Control: CARE-induced rats were treated with distilled water VitE: CARE-induced rats were treated with vitamin E 30 mg/kg body weight; SC100: CARE-induced rats were treated with SC 100 mg/kg body weight; SC200: CARE-induced rats were treated with SC 200 mg/kg body weight. Data are mean ± SD (*n* = 8). Significance: ^###^*P* < 0.001 vs. normal group and ^*∗*^*P* < 0.05, ^*∗∗*^*P* < 0.01, ^*∗∗∗*^*P* < 0.001 vs. control group.

Involved in the degradation of an extracellular matrix, MMP/TIMP proteins are also involved in various physiological and pathological processes in inflammatory diseases. The expressions of MMPs were increased in the Control group compared with the Normal group (MMP-2, 1.45-fold; MMP-9, 1.31-fold), whereas they were significantly decreased in the SC-treated rats compared with the Control group. In particular, MMP levels were reduced to below that of the Normal group by the SC-treated. Conversely, the expressions of TIMPs were decreased in the Control group compared with the Normal group (TIMP-1, 0.47-fold; TIMP-2, 0.43-fold), and SC treatment significantly increased levels of TIMPs (Figure 6).

### 3.9. Histological Examination of Esophagus

**Figure 7:**
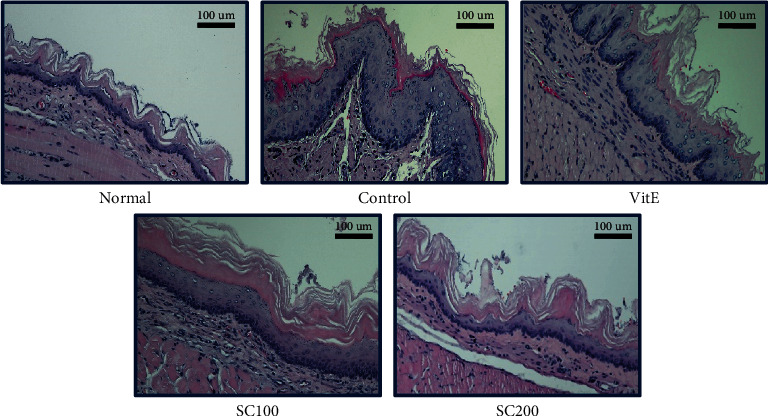
Histological examination of esophagus through H&E staining. Magnification ×200. Normal: normal group; Control: CARE-induced rats were treated with distilled water; VitE: CARE-induced rats were treated with vitamin E 30 mg/kg body weight; SC100: CARE-induced rats were treated with SC 100 mg/kg body weight; SC200: CARE-induced rats were treated with SC 200 mg/kg body weight.

The mucosal state of the esophageal tissue was confirmed through H&E staining. The esophagus in the Normal group was normal without the infiltration of inflammatory cells. However, the Control group showed the elimination of the mucosa and exhibited considerable inflammatory cell infiltration. In the SC100 and the SC200 groups, it was confirmed that the damage was alleviated by protecting the esophageal mucosa through SC treatment (Figure 7).

## 4. Discussion

The anti-inflammatory effects of Scutellariae Radix and Citri Reticulatae Pericarpium are well known in modern medicine, and various studies related to inflammation have been conducted [22, 29, 30]. Plant specimens have been used for various diseases for a long time, and various studies using plant specimens are being conducted [31–33]. Plant specimens contain various components such as alkaloids and flavonoids, and it has been reported that these components exhibit various pharmacological effects [34]. As a result of HPLC analysis, it was confirmed that various flavone components such as baicalin 148 mg/g, baicalein 12 mg/g, narirutin 12 mg/g, and hesperidin 10 mg/g were also contained in Scutellariae Radix and Citri Reticulatae Pericarpium used in this experiment, and these ingredients are expected to help alleviate various diseases by showing excellent antioxidant effects. In this study, the effect on reflux esophagitis was confirmed by mixing Scutellariae Radix and Citri Reticulatae Pericarpium, which contain various flavone components and are known to have an excellent effect on inflammation. As a result of previous studies, it was confirmed that the Scutellariae Radix and Citri Reticulatae Pericarpium mixture (SC) alleviated the damage to the esophageal mucosa in acute reflux esophagitis, and in this experiment [16], the effect of SC on chronically advanced reflux esophagitis and its mechanism were confirmed. SC treatment significantly reduced the degree of esophageal mucosal damage, significantly reduced levels of MDA and MPO, and inhibited the activation of the NF-*κ*B inflammatory pathway by activating the PPAR*γ*/RXR pathway. In addition, SC treatment modulated the MMP/TIMP proteins.

In recent studies, oxidative stress is considered the cause of various inflammations [35–37], and it has been found that reflux esophagitis is also closely related to oxidative stress [38, 39]. In reflux esophagitis, oxidative stress induces the depletion of the esophageal mucosa and plays an important role in damaging the esophageal mucosa from acid secreted by parietal cells [40]. Oxidative stress is caused by excessive activation of NADPH oxidase and promotion of oxidation, and as a result of this process, an increase in malondialdehyde (MDA), an important product of lipid peroxidation, and an increase in myeloperoxidase (MPO), an indicator of tissue neutrophil infiltration, are induced. Also, MDA and MPO are considered an indicator of mucosal damage caused by oxidative stress [41, 42]. In this study, the stimulation of NADPH oxidase (NOX2 and p47^phox^) and MDA and MPO increased due to reflux esophagitis, whereas SC treatment significantly reduced the index related to increased oxidative stress. These results suggest that SC can effectively reduce oxidative stress.

Peroxisome proliferator-activated receptor *γ* (PPAR*γ*) is a transcription factor widely involved in metabolism, development, differentiation, and tumorigenesis, forms a homodimers or heterodimers with retinoid *X* receptor (RXR), and is considered a promising candidate for the treatment of various diseases [43, 44]. Also, it is mainly present in endothelium, macrophages, and monocytes and is known to suppress the expression of cytokines related to inflammation by inhibiting NF-*κ*B in immune and inflammatory responses [45]. In addition, Porvani et al. reported that PPAR*γ* directly regulates the expression of antioxidant genes through interaction with oxidative stress-related signaling pathways and exerts beneficial effects on cellular damage caused by reactive oxygen species [7, 46]. As a result of this experiment, it was confirmed that SC treatment activated the PPAR*γ*/RXR pathway and increased the expression of antioxidant enzymes such as SOD-1 and catalase in reflux esophagitis. In addition, SC treatment inhibited NF-*κ*B pathway through the activation of the PPAR*γ*/RXR pathway, thereby reducing the expression of inflammation-related cytokines.

Cyclooxygenase enzymes such as COX-1 and COX-2 catalyze the production of prostaglandin (PG) from arachidonic acid [47]. COX-1 protects the gastrointestinal mucosa and maintains the integrity of the stomach, and COX-2 is rapidly induced in the inflammatory site of the stomach and colon to participate in the inflammatory response [48]. Here, the inhibition of COX-1 induces the upregulation of COX-2, and the upregulated COX-2 is involved in various acute and chronic inflammations, and thus is considered an important target of many pharmacological inhibitors [49]. Among the PG produced in this process, type E (PGE) is the most effective in regulating various functions of the digestive tract, such as gastric cell protection in reflux esophagitis, and plays an important mediator role in the inflammatory process [47, 50]. As a result of this experiment, SC treatment significantly regulated the expression of COX-1 and COX-2 and also significantly decreased the expression of PGE_2_ in reflux esophagitis.

Matrix metalloproteinases (MMPs) and tissue inhibitor of metalloproteinases (TIMPs) are related to the extracellular matrix (ECM) and are involved in tumor progression, invasion, and tissue remodeling [51]. Overexpression of MMP in the esophagus can induce the development and progression of esophageal cancer [52]. It has been reported that the overexpression of MMP-2 can promote the development of malignant tumors, and in a study by Wang et al., the activation of the NF-*κ*B signaling pathway upregulates the expression of MMP-2 [53]. As a result of this experiment, SC treatment helped to achieve balanced expression by regulating the expression of MMPs and TIMPs.

## 5. Conclusions

In conclusion, in the present study, the treatment of the SC significantly reduced oxidative stress-related factors. Also, the anti-inflammatory effects of the SC suggested the inhibition of NF-*κ*B pathway through the activation of the PPAR*γ*/RXR pathway, thereby reducing the expression of inflammation-related cytokines. In addition, the treatment of the SC significantly modulated MMP/TIMP proteins. Consequently, SC improved the damage to the esophageal mucosa. Taken together, SC not only regulates the expression of antioxidant-related factors by reducing oxidative stress, but also inhibits the NF-*κ*B pathway through activation of the PPAR*γ*/RXR pathway, thereby alleviating inflammation of the esophageal mucosa. These results help to understand the effect and mechanism of SC on reflux esophagitis.

## Figures and Tables

**Table 1:** Levels of MDA and MPO.

	MDA		MPO (mU/mL)
	Serum (nmol/mL)	Tissue (nmol/mg protein)	
Normal	2.27 ± 0.23	1.65 ± 0.11	1,852 ± 134
Control	6.37 ± 1.14^###^	2.99 ± 0.38^###^	2,849 ± 53^###^
VitE	3.37 ± 0.70^*∗∗*^	1.80 ± 0.11^*∗∗∗*^	2,500 ± 108^*∗*^
SC100	2.00 ± 0.13^*∗∗∗*^	1.93 ± 0.07^*∗∗*^	2,372 ± 123^*∗∗*^
SC200	1.90 ± 0.19^*∗∗∗*^	1.90 ± 0.14^*∗∗*^	2,308 ± 58^*∗∗*^

Normal: normal group; Control: CARE-induced rats were treated with distilled water; VitE: CARE-induced rats were treated with vitamin E 30 mg/kg body weight; SC100: CARE-induced rats were treated with SC 100 mg/kg body weight; SC200: CARE-induced rats were treated with SC 200 mg/kg body weight. Data are mean ± SD (*n* = 8). Significance: ^###^*P* < 0.001 vs. normal group and ^*∗*^*P* < 0.05, ^*∗∗*^*P* < 0.01, ^*∗∗∗*^*P* < 0.001 vs. control group.

## Data Availability

The datasets used and analyzed during this work are available from the corresponding author upon reasonable request.

## Supplementary Materials

Supplementary Materials Supplementary material 1: HPLC profile of Scutellariae Radix and Citri Reticulatae Pericarpium. HPLC profile of baicalin and baicalein in Scutellariae Radix (A); HPLC profile of narirutin and hesperidin in Citri Reticulatae Pericarpium (B).
