# Supplementary figures and images for: Scutellariae Radix and Citri Reticulatae Pericarpium Mixture Regulate PPARγ/RXR Signaling in Reflux Esophagitis
Source: Evid Based Complement Alternat Med. 2022 Jan 4;2022:6969241. doi: 10.1155/2022/6969241 (PMC8752236; doi:10.1155/2022/6969241)

(A)

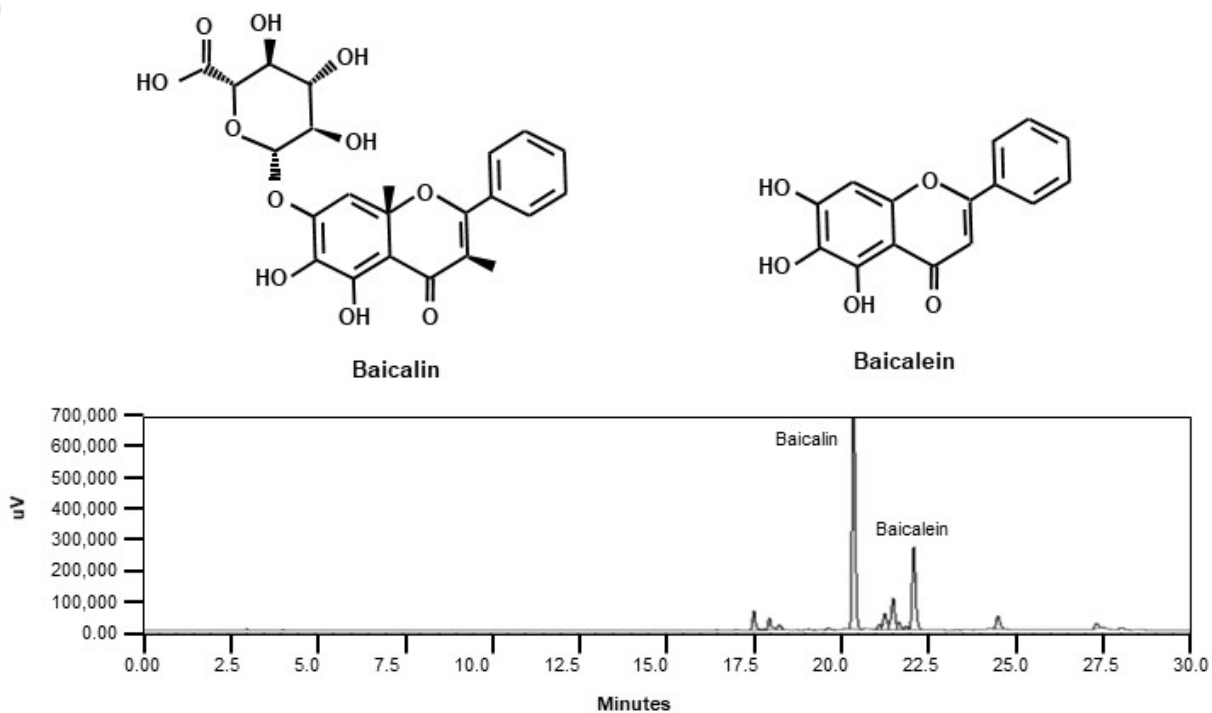

(B)

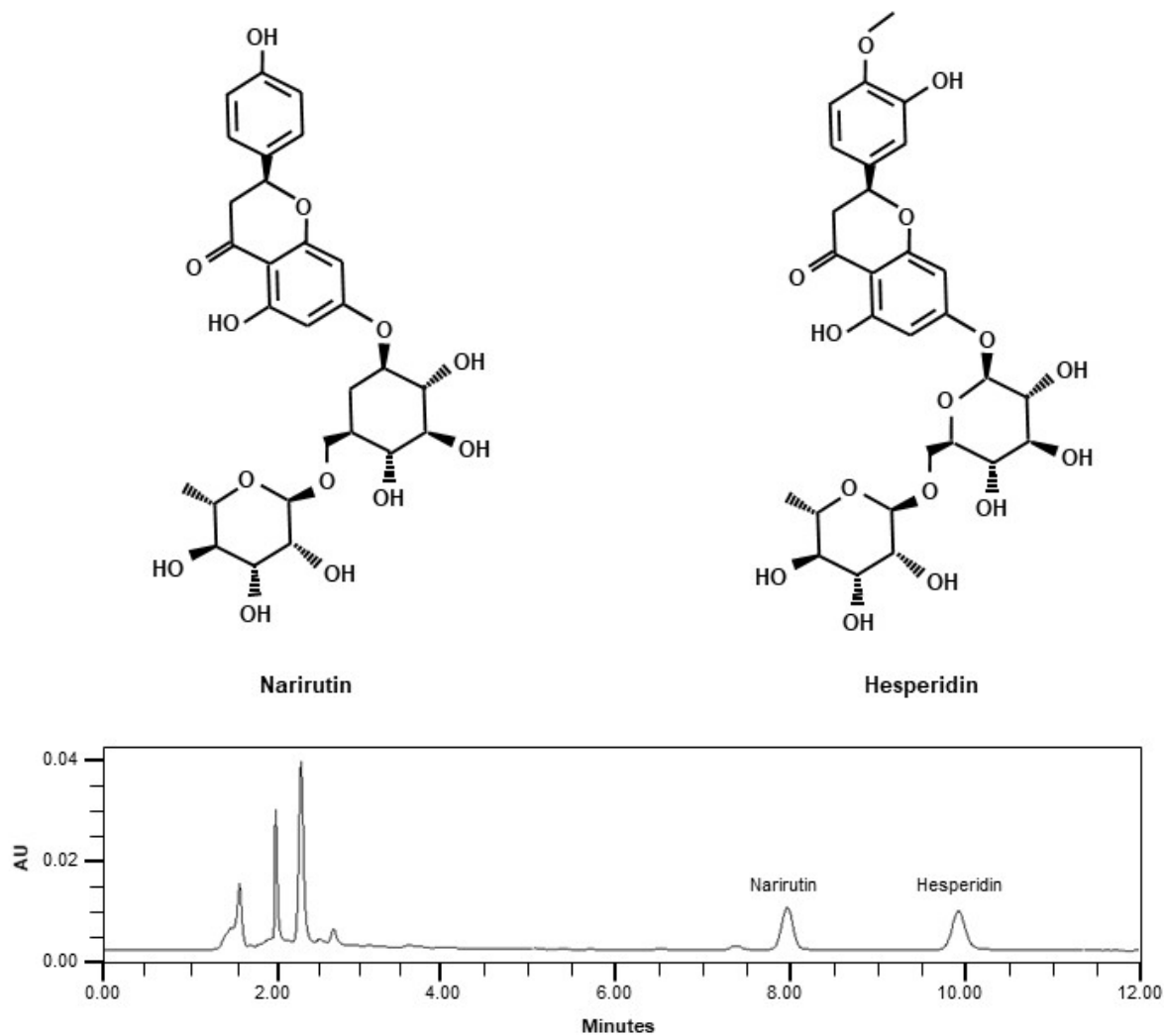

Supplement: Supplementary Materials — Supplementary material 1: HPLC profile of Scutellariae Radix and Citri Reticulatae Pericarpium. HPLC profile of baicalin and baicalein in Scutellariae Radix (A); HPLC profile of narirutin and hesperidin in Citri Reticulatae Pericarpium (B). [file 6969241.f1.pdf]
